# Poor Regenerative Outcome after Skeletal Muscle Necrosis Induced by *Bothrops asper* Venom: Alterations in Microvasculature and Nerves

**DOI:** 10.1371/journal.pone.0019834

**Published:** 2011-05-24

**Authors:** Rosario Hernández, Carmen Cabalceta, Patricia Saravia-Otten, Alessandra Chaves, José María Gutiérrez, Alexandra Rucavado

**Affiliations:** 1 Facultad de Ciencias Químicas y Farmacia, Universidad de San Carlos de Guatemala, Finca San Isidro, Guatemala; 2 Instituto Clodomiro Picado, Facultad de Microbiología, Universidad de Costa Rica, San José, Costa Rica; The University of Hong Kong, Hong Kong

## Abstract

**Background:**

Viperid snakebite envenoming is characterized by prominent local tissue damage, including muscle necrosis. A frequent outcome of such local pathology is deficient skeletal muscle regeneration, which causes muscle dysfunction, muscle loss and fibrosis, thus provoking permanent sequelae that greatly affect the quality of life of patients. The causes of such poor regenerative outcome of skeletal muscle after viperid snakebites are not fully understood.

**Methodology/Principal Findings:**

A murine model of muscle necrosis and regeneration was adapted to study the effects of the venom and isolated toxins of *Bothrops asper*, the medically most important snake in Central America. Gastrocnemius muscle was injected with either *B. asper* venom, a myotoxic phospholipase A_2_ (Mtx), a hemorrhagic metalloproteinase (SVMP), or saline solution. At various time intervals, during one month, tissue samples were collected and analyzed by histology, and by immunocytochemical and immunohistochemical techniques aimed at detecting muscle fibers, collagen, endothelial cells, myoblasts, myotubes, macrophages, TUNEL-positive nuclei, and axons. A successful regenerative response was observed in muscle injected with Mtx, which induces myonecrosis but does not affect the microvasculature. In contrast, poor regeneration, with fibrosis and atrophic fibers, occurred when muscle was injected with venom or SVMP, both of which provoke necrosis, microvascular damage leading to hemorrhage, and poor axonal regeneration.

**Conclusions/Significance:**

The deficient skeletal muscle regeneration after injection of *B. asper* venom is likely to depend on the widespread damage to the microvasculature, which affects the removal of necrotic debris by phagocytes, and the provision of nutrients and oxygen required for regeneration. In addition, deficient axonal regeneration is likely to contribute to the poor regenerative outcome in this model.

## Introduction

Snakebite envenomings constitute a highly relevant and neglected public health problem on a world wide basis, affecting particularly the rural settings of Latin America, Africa and Asia [1]–[3]. In Latin America, the majority of snakebites are inflicted by species classified in the genus *Bothrops* [4], [5]. These envenomings are characterized by complex pathological and pathophysiological profiles that include prominent local tissue damage, i.e. necrosis, hemorrhage, blistering and edema, and systemic alterations, i.e. bleeding, coagulopathy, cardiovascular shock and renal failure [6]–[10]. Among these effects, local tissue damage leading to necrosis is particularly relevant, since it is frequently followed by poor tissue regeneration, with the occurrence of permanent sequelae associated with tissue loss and dysfunction, and their consequent social and psychological implications [1], [11], [12].

Skeletal muscle regeneration is a complex and finely orchestrated process that involves the interaction of myogenic cells, other resident cells, inflammatory cells, blood vessels, nerves and extracellular matrix [13]. It starts after the injury of muscle fibers, with the activation of a population of quiescent myogenic cells, satellite cells, located at the periphery of the fibers between muscle sarcolemma and the basement membrane [13]–[15]. Once activated, satellite cells become myoblasts which, after various cycles of replication, undergo a process of cell fusion leading to the formation of multinucleated myotubes which then mature to become adult myofibers [16]. For this process to proceed successfully, several requirements must be met in the microenvironment where regeneration takes place. After muscle fiber necrosis, an inflammatory reaction ensues, with invasion of the tissue by neutrophils and macrophages. Besides removing necrotic debris, these cells, especially macrophages, synthesize various cytokines and growth factors which modulate the tissue regenerative response, and also support myoblast survival in various ways [17]. Furthermore, an intact microvascular supply is required for regeneration [13], together with a restitution of the neuromuscular junction in the regenerative fibers [18]. The persistence of a structurally intact basement membrane around necrotic muscles is also important for the demarcation of the space where myoblast replication and fusion occurs [13], [19]. Various muscle pathologies are associated with a good regenerative outcome, especially in conditions where blood supply and innervation are not affected, whereas in other diseases, such as ischemic lesions and some muscular dystrophies, regeneration is deficient [13], [20]. Unveiling the factors associated with deficient muscle regeneration in various diseases, including snakebite envenoming, is highly relevant for the design of interventions aimed at improving this process.

In the case of snake venom-induced myonecrosis, two clearly different patterns of regeneration have been described: when tissue is affected by venoms or toxins that induce muscle necrosis but do not affect the integrity of blood vessels, such as after injection of isolated myotoxic phospholipases A_2_ (PLA_2_) or cardiotoxins, regeneration proceeds successfully [21]–[23]. Some of these myotoxins have been used as models to investigate particular aspects of muscle regeneration [21]. In contrast, when muscle is affected by venoms which, in addition to affecting muscle fibers also damage the microvasculature, inducing hemorrhage, such as many viperid snake venoms, the regenerative process is impaired, with substitution of muscle tissue by fibrosis in some areas, and with the presence of regenerating fibers of reduced diameter [22], [24]–[26]. In the case of *Bothrops* sp venoms, these experimental observations constitute an excellent correlate of the clinical situation, where tissue loss and dysfunction are common consequences of these envenomings [6], [7], [9].

From the medical standpoint *Bothrops asper* is the most important snake in southern Mexico, Central America and northern areas of South America [5], [27]. Envenomings by this species are associated with local tissue damage which often leads to sequelae associated with impaired muscle regeneration [6], [7], [9]. Such phenomenon has been investigated at the experimental level, and it has been proposed that microvascular damage provoked by hemorrhagic snake venom metalloproteinases (SVMPs) is a key factor in the poor regenerative outcome [22], [24], [28]. However, the majority of these studies have been based on a qualitative histological assessment of tissue alterations; moreover, other factors, such as damage to nerves and myogenic cells, have not been studied. It is necessary, therefore, to further investigate this phenomenon from a quantitative perspective, and to assess the participation of various factors involved in tissue damage and repair, in order to identify the critical processes being affected that preclude a successful regenerative outcome. This study examined, using a combination of qualitative and quantitative approaches, the skeletal muscle regeneration in murine skeletal muscle injected with the venom of *B. asper*, and with some purified toxins. It was found that critical events occur within the first days after tissue necrosis, with microvascular damage occupying a central role in the stage. In addition, a poor regeneration of axons is described, which is likely to affect muscle regeneration. The characterization of this deficient regenerative scenario may become a useful experimental model to assess interventions aimed at improving the process of muscle regeneration in this and related pathologies.

## Methods

### Animals, venoms and toxins

Mice (CD1, 18–20 g body weight) were used throughout the study. Venom was obtained from a collection of adult *B. asper* specimens from the Pacific versant of Costa Rica, maintained at the serpentarium of Instituto Clodomiro Picado. Immediately after collection, venom was lyophilized and stored at −20°C. The hemorrhagic SVMP BaP1 was isolated from this venom, as previously described [29], [30]. A myotoxic fraction (Mtx) was isolated from this venom by ion-exchange chromatography on CM-Sephadex C-50 [31]. This fraction was a mixture of the two types of basic myotoxins present in the venom, i.e. a catalytically-active Asp49 PLA_2_ and a catalytically-inactive PLA_2_ homologue, in a 1∶2 approximate molar ratio, which roughly corresponds to the proportion of these types of myotoxins in the venom [32]. This myotoxic fraction was utilized in order to reproduce the combined action of these molecules in the crude venom, since a synergistic effect has been described for these Asp49 and Lys49 myotoxic PLA_2_s [33]. Venom and toxin solutions were prepared in 0.14 M NaCl, 0.04 M phosphate, pH 7.2, solution (PBS) immediately before use.

### Ethics statement

The experimental protocols involving the use of animals in this study were approved by the Institutional Committee for the Care and Use of Laboratory Animals (CICUA) of the University of Costa Rica (protocol CICUA-27-10) and adhere to the International Guiding Principles for Biomedical Research Involving Animals of the Council of International Organizations of Medical Sciences (CIOMS).

### Histological analysis

Mice were injected intramuscularly, in the right gastrocnemius, with either crude venom (50 µg), Mtx (100 µg), or BaP1 (50 µg), dissolved in 50 µl of PBS. These doses were selected since they induce a similar extent of tissue damage [22], [24]. Muscles injected with PBS under otherwise identical conditions were used as controls. For histological analysis, mice were sacrificed at different times (1 and 6 hr, and 1, 3, 7, 14 and 28 days) and the injected gastrocnemius muscles were dissected out and placed in a zinc fixative solution (BD Pharmigen, USA), for at least 48 hours at 4°C. These time intervals were selected because they cover the acute phase of tissue damage (1 and 6 hr), the inflammatory reaction following necrosis (1 and 3 days), and the various steps in the process of muscle regeneration (3, 7, 14 and 28 days) [22], [24]. The tissue was then dehydrated in ethanol, placed in xylene, and embedded in paraffin using conventional protocols [34]. For quantitative assessment of tissue changes, three non-consecutive sections of 4 µm were obtained from the mid-belly region of each muscle, placed in glass slides, deparaffinized in xylene, hydrated in distilled water and stained with hematoxylin and eosin for microscopic evaluation. Three images were captured from each section using an Evolution MP camera (Media Cybernetics, USA). The extent of necrosis was estimated in samples collected 24 hr after injection as the percentage of the examined area corresponding to necrotic fibers, which were identified by characteristic morphological alterations, i.e. hypercontraction or coagulative necrosis. The extent of regeneration was estimated in samples collected at 28 days as the percentage of the examined area corresponding to regenerating fibers, i.e. fibers having a centrally-located nuclei. In addition, the diameters of regenerating fibers in tissue samples collected 28 days after injection were determined. Both types of analyses were performed using an image analysis software (Image Pro 6.3, Media Cybernetics, USA).

### Sirius Red staining of collagen

Groups of three mice were injected in the right gastrocnemius muscle with either PBS, venom, Mtx or BaP1, at the doses described above, and sacrificed 3, 5 and 7 days after treatment. In order to detect fibrosis, i.e. increments in collagen in the tissue, sections were stained with Sirius Red dye CI 35780 (Polyscience Inc, USA), which stains collagen. The procedure described by Montes and Junqueira [35] was followed, with some modifications. In brief, sections were stained with picro Sirius red (0.1% in a saturated picric acid solution) and Fast Green FCF 0.1% (for staining proteins) (Sigma, USA), for one hr at room temperature. The slides were washed twice with acidified water (5 ml of glacial acetic acid per liter), dehydrated, cleared in xylene and mounted (Shandon Xylene Substitute Mountant, Thermo Scientific, USA).

### Changes in the capillary density and the capillary/muscle fiber ratio

Groups of three mice were injected in the right gastrocnemius muscle with crude venom (50 µg), dissolved in 50 µl of PBS, and sacrificed at various time intervals covering the degenerative and the regenerative phases (1 and 6 hr, and 1, 3, 7, 14 and 28 days). In some experiments, groups of mice were injected with 100 µg Mtx, and sacrificed at 24 hr and 28 days. Control muscles were obtained from groups of mice injected with 50 µl of PBS at all time intervals studied. Muscles were dissected out and processed as described above. Three non-consecutive sections of 4 µm were obtained per muscle, placed in positive charged glass slides (Erie Scientific, USA), deparaffinized and hydrated. The detection of capillary vessels was performed by fluorescence immunohistochemistry. Antigen retrieval was carried out by incubating tissue sections with Proteinase K (Dako, USA) for 5 min at room temperature. Blockage steps were performed with avidin and biotin solutions (Biotin blocking system, Dako Cytomation, USA), according to the manufacturer's instructions, as well as with rabbit immunoglobulins, for 10 min each. Then, sections were incubated with rat anti-mouse CD31 monoclonal antibody (Cymbus Biotechnology, USA), at 1∶100 dilution for one hr at room temperature. After washing with PBS, sections were incubated with polyclonal biotinylated rabbit anti-rat IgG (Dako Cytomation, Denmark), at 1∶200 dilution for one hr at room temperature. The reaction was amplified using the Tyramide amplification kit (Perkin Elmer, USA), and finally sections were incubated with a 1∶500 dilution of Streptavidin Alexa fluor 488 for one hr at room temperature. Nuclear staining was performed with DAPI (4′, 6′ –diamino-2-phenylindole, Invitrogen, USA). Three images, from randomly selected non-overlapping areas, were captured per section. The total number of capillary vessels, the number of muscle fibers and the total area of the section were quantified in each image using the image analysis software described above. The number of muscle fibers counted per image ranged between 70 and 150, and the total number of muscle fibers counted in the three images corresponding to each tissue section ranged between 250 and 300. Then, the capillary / muscle cell ratio and the number of capillaries per area were calculated.

### Immunostaining for Pax7 and desmin

Groups of three mice were injected in the right gastrocnemius with either PBS, venom or Mtx, at the doses described above, and sacrificed 3, 5 and 7 days after treatment. The identification of myogenic cells was performed by immunohistochemistry using antibodies against Pax7 (to detect activated satellite cells/myoblasts at 3 days) or against desmin (to detect myotubes and myofibers at 3, 5 and 7 days). Briefly, antigen retrieval was carried out using citrate buffer, pH 6.0, and heating in microwave oven for 5 min. Primary mouse anti-human Pax7 antibody (Abcam, USA), or mouse anti-human desmin antibody (Dako Cytomation, Denmark) were used at 1∶100 dilution and incubated for one hr at room temperature. Then, sections were incubated with polyclonal biotinylated goat anti-mouse IgG (Dako Cytomation, Denmark), at 1∶200 dilution for one hr at room temperature. Sections were then incubated with a 1∶500 dilution of Streptavidin Alexa fluor 488 for one hr at room temperature. Three images, from non-overlapping areas affected by the treatments, i.e. showing necrotic fibers, fibrosis or regenerating fibers, were captured per muscle section.

### Evaluation of TUNEL-positive nuclei in regenerating muscle fibers

Groups of three mice were injected in the right gastrocnemius muscle with either PBS, venom or Mtx, at the doses described above, and sacrificed 3, 5 and 7 days after treatment. These time intervals were selected because critical steps involving inflammatory and regenerative events occur during these days in experimental models of acute muscle damage [13], [21]. Three non-consecutive sections of 4 µm were obtained per muscle, and were evaluated for the presence of TUNEL-positive cells by using the ApopTag® Red In Situ Apoptosis Detection Kit (Millipore, USA), following the protocol described by the manufacturer. Sections were also immunostained for either Pax7 or desmin, as described above, to detect activated satellite cells or myotubes and regenerating myofibers, respectively. Pax7-positive and desmin-positive cells containing TUNEL-positive nuclei were quantified.

### Quantification of nerves in muscle tissue

Groups of five mice were injected in the right gastrocnemius muscle with either PBS, venom, Mtx or BaP1, at the doses described above. Animals were sacrificed 3 and 28 days after injection, in order to detect changes in the density of intramuscular nerves after the degenerative and regenerative phases, respectively. Three non-consecutive sections of 4 µm were obtained per muscle, placed in positive charged glass slides (Erie Scientific, USA), deparaffinized and hydrated. The immunohistochemical detection of nerves was performed using a mouse anti-human neurofilament protein (Dako, Denmark). The staining protocol was similar to that described for Pax7 and desmin, except for the primary antibody. Structures of 10–40 µm diameter showing internal fibers positively stained for neurofilament protein were identified as intramuscular nerves. The total number of nerves and the area of muscle tissue examined were determined, in order to estimate the number of nerves per area. In addition, the number of axons within nerves was quantified and the axonal density per area of nerve was determined. Both analyses were performed using the image analysis software described.

### Statistical analysis

Data were analyzed by the statistics software SPSS 15.0. Means, variance and standard deviation were calculated for each parameter and treatment, and compared using the Friedman test. When appropriated, an additional Wilcoxon test was carried out.

## Results

### Muscle regeneration is deficient in tissue affected by hemorrhage

**Figure 1:**
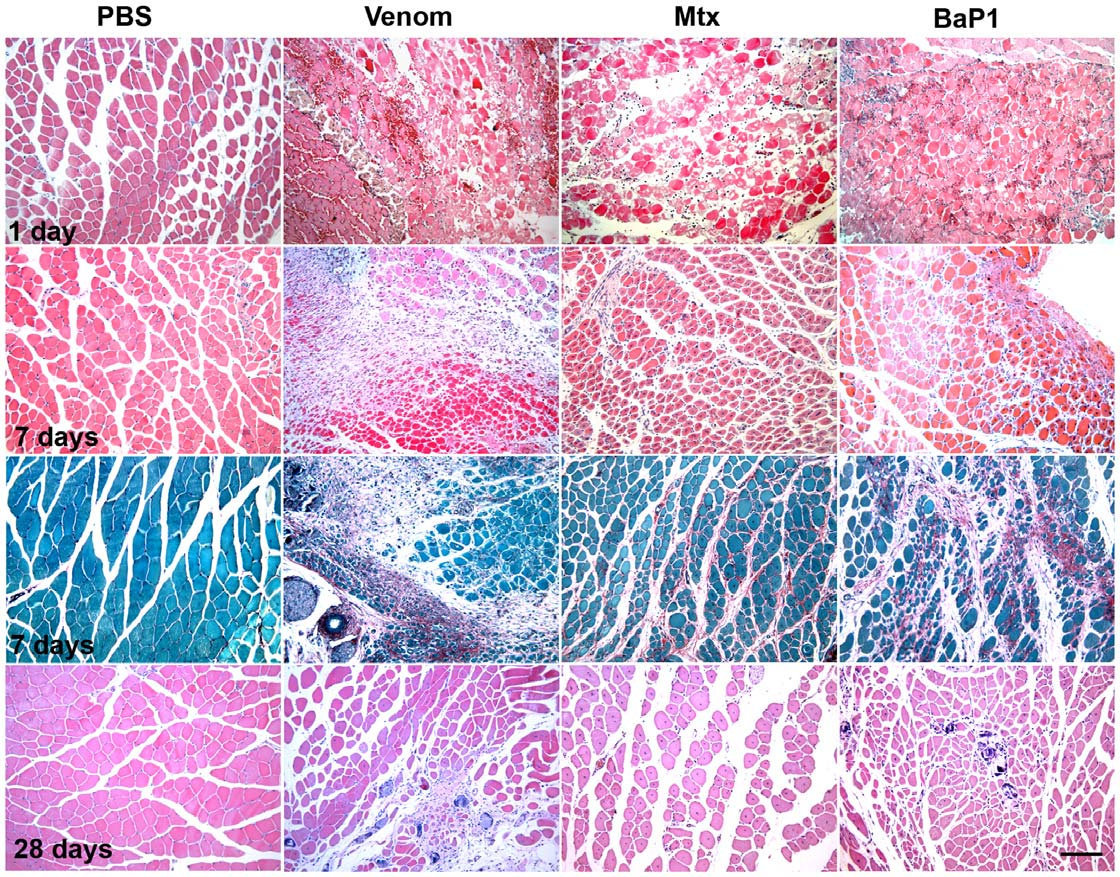
Light micrographs of sections of mouse skeletal muscle at 1, 7 and 28 days after the injection, in the gastrocnemius muscle, of phosphate-buffered saline solution (PBS), *B. asper* venom, Myotoxin (Mtx), and metalloproteinase BaP1. First, second and fourth horizontal rows of figures correspond to hematoxylin-eosin-stained sections, whereas the third row corresponds to sections stained with Sirius Red and counterstained with Fast Green FCF. Bar represents 100 µm.

Histological observations showed that all treatments, except PBS, induced evident myonecrosis in the injected gastrocnemius one day after injection (Fig 1). In the case of muscle injected with venom or BaP1, hemorrhage was also observed, whereas no hemorrhage was induced by Mtx. An inflammatory infiltrate was present in muscles injected with venom or toxins by 1 and 3 days. Small regenerating fibers, characterized by centrally-located nuclei, were present at 3 days and thereafter, although differences were noticed among the various treatments. In the case of muscle injected with Mtx, by 7 days, regenerating fibers were distributed uniformly in the tissue (Fig 1). In contrast, muscle injected with venom or BaP1 showed areas of regenerating fibers intermixed with areas of remnants of necrotic cells, which had very low numbers of phagocytes, and areas of fibrosis (Fig 1). Therefore, the histological pattern at 7 days in muscle injected with venom was characterized by heterogeneity, whereas in the case of tissue affected by Mtx, a homogeneous pattern predominated (Fig 1). Fibrosis was corroborated 7 days after injection, by staining with Sirius Red, in the endomysium and perimysium in muscle injected with venom or BaP1 (Fig 1), whereas the extent of collagen deposition was less evident in Mtx-injected muscle, being nevertheless higher than in PBS-injected muscle. By 28 days, tissue injected with Mtx presented a pattern of successful regeneration, with abundant regenerating fibers of similar size having centrally-located nuclei, and with little fibrosis, albeit with an increment in the interstitial area as compared with PBS-injected muscle (Fig 1). In contrast, in the case of muscle injected with venom or BaP1, which induced hemorrhage, regenerating fibers of small size were observed, and were intermixed with areas of fibrosis and, in some cases, with areas in which the remnants of necrotic fibers had apparently become calcified (Fig 1).

**Figure 2:**
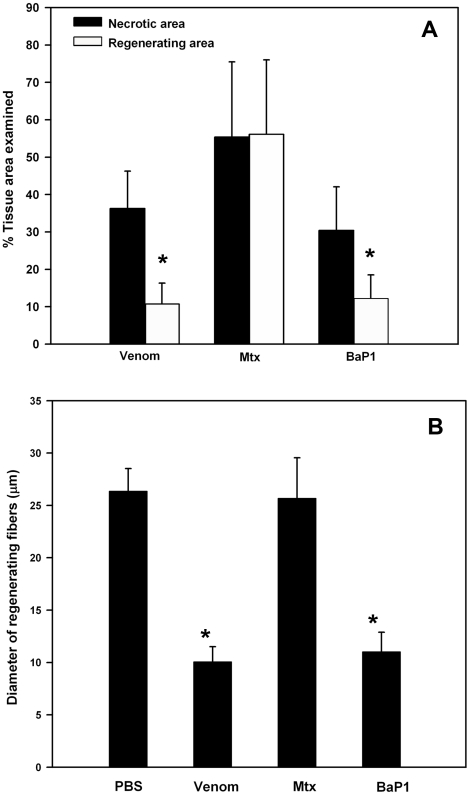
(A) Quantitative assessment of the extent of myonecrosis and regeneration in mouse gastrocnemius muscle injected with either *B. asper* venom, Mtx or BaP1. Histological sections stained with hematoxylin and eosin were analyzed one and 28 days after injections. The extent of necrosis was estimated in samples collected one day after injection as the percentage of the examined area corresponding to necrotic fibers, whereas the extent of regeneration was estimated in samples collected at 28 days as the percentage of the examined area corresponding to regenerating fibers, i.e. fibers having centrally-located nuclei. *p < 0.05 when comparing the percentage of necrosis and of regeneration for a single treatment. (B) Quantitative assessment of the diameter of regenerating muscle fibers, i.e. fibers presenting centrally-located nuclei, 28 days after intramuscular injection in the gastrocnemius of PBS, *B. asper* venom, Mtx or BaP1. Regenerating fibers in tissue injected with venom and BaP1 showed a reduced diameter when compared with control fibers in tissue injected with PBS (p < 0.05), whereas no significant difference was observed in the diameter of regenerating fibers in muscle injected with Mtx (p > 0.05). In both graphs, results are presented as mean±SD (n = 9).

The extent of muscle necrosis and regeneration was quantitatively assessed by measuring the area corresponding to necrotic fibers in gastrocnemius muscle one day after injection of venom or isolated toxins, and the area of regenerating fibers, i.e. fibers with centrally located nuclei, 28 days after injection. As shown in Fig 2, all treatments induced acute muscle damage which ranged from 30% necrotic muscle in the case of SVMP BaP1 to 55% necrotic muscle in tissue injected with of Mtx. Muscle regeneration was significantly affected in the case of treatments that induced hemorrhage besides local myonecrosis, i.e. venom and BaP1 (Fig 2A). In contrast, a successful regeneration was observed in tissue injected with Mtx alone (Fig 2A). In addition, the diameter of regenerative fibers 28 days after injection greatly differed among the various treatments. The diameters of regenerating fibers in tissue injected with Mtx did not differ from those of control, PBS-injected muscle fibers (Fig 2B). In contrast, the diameter of regenerating fibers in tissue treated with either venom or BaP1, both of which induced hemorrhage, were smaller than those of control fibers (Fig 2B). On the basis of these results, the rest of the study was focused mostly on the comparison of two experimental groups: muscle injected with venom, as a model of poor regeneration, and muscle injected with Mtx, as a model of successful regeneration.

### *B. asper* venom induces rapid damage to capillary vessels followed by revascularization

**Figure 3:**
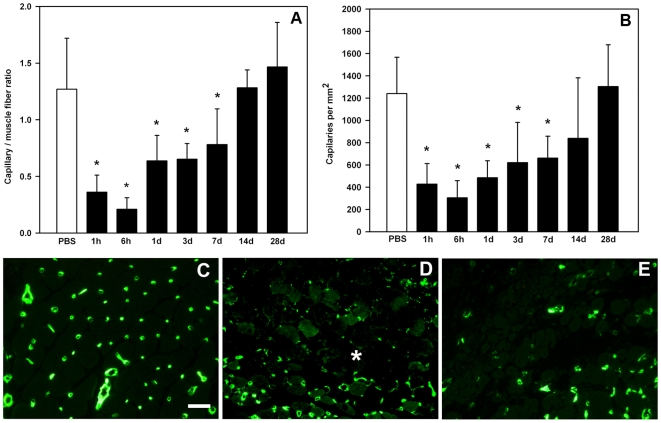
Time-course of changes in capillary/muscle fiber ratio (A) and in capillary density (capillaries per mm^2^, B) in mouse gastrocnemius muscle injected with *B. asper* venom; control mice were injected with PBS. Capillaries were visualized by immunohistochemistry with a rat anti-mouse CD31 monoclonal antibody, as described in Materials and Methods. A reduction in both parameters of capillary density occurred rapidly after venom injection, followed by a revascularization process. Results are presented as mean±SD (n = 9). * p < 0.05 when compared with capillary/muscle cell ratio and capillary density of muscle injected with PBS. (C–E) Immunohistochemical staining of endothelial cells in sections of mouse gastrocnemius muscle one day after injection of PBS (C), or one day (D) and seven days (E) after injection of *B. asper* venom. Endothelial cells were detected with anti-CD31 antibody, followed by a polyclonal biotinylated rabbit anti-rat IgG and Streptavidin Alexa fluor 488 (see Methods for details). Notice the loss of endothelial cell immunostaining in some areas in muscle injected with venom (asterisk). Bar represents 50 µm.

Injection of *B. asper* venom, at a dose that induces prominent hemorrhage, resulted in a drastic and rapid drop in the number of capillary vessels in gastrocnemius muscle, as judged both by the capillary / muscle fiber ratio and by the number of capillaries per tissue area. Both parameters of capillary density remained significantly reduced, when compared with control values, during the first week (Fig 3A and B). Muscle injected with PBS had a normal pattern of capillaries, as evidenced by immunostaining for CD31 (Fig 3C). A spatially heterogeneous pattern of capillary damage was observed rapidly after venom injection (Fig 3D). The number of capillaries increased by 7 days (Fig 3A, B and E) and reached values similar to those of control tissue by 14 and 28 days (Fig 3A and B), thus evidencing a revascularization process. In contrast, when Mtx was administered, the number of capillaries was not affected (results not shown), in agreement with the lack of hemorrhagic activity of this toxin.

### A spatially heterogeneous pattern of regenerating fibers with TUNEL-positive nuclei was observed in muscle treated with venom

**Figure 4:**
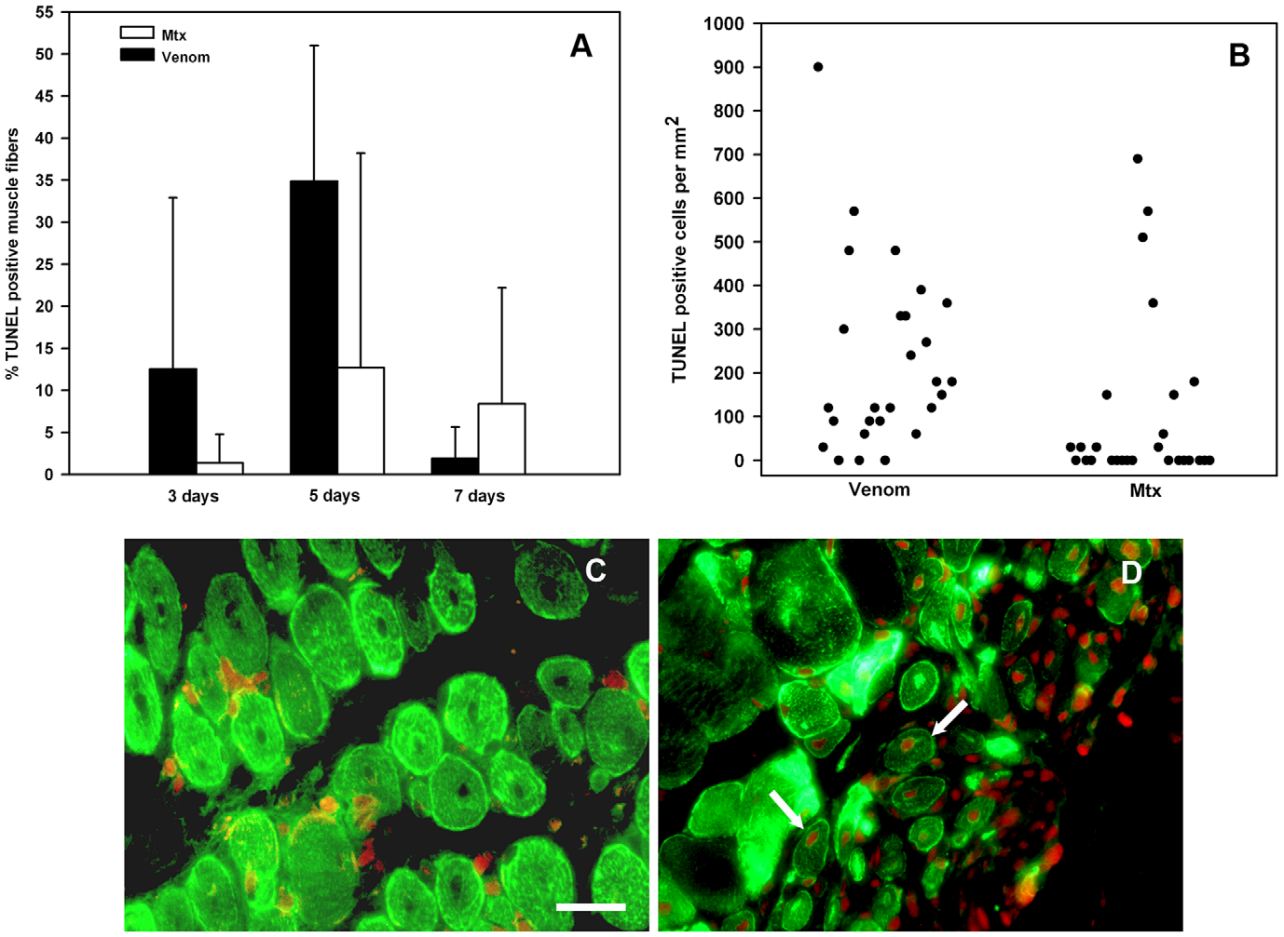
Apoptosis in regenerating skeletal muscle after the injection of either *B. asper* venom or Mtx. Sections from muscle tissue samples collected 3, 5 and 7 days after injection of venom or Mtx were immunostained with anti-desmin antibodies and with TUNEL (see Methods for details). (A) The percentage of desmin-positive regenerating muscle fibers showing at least one TUNEL-positive nuclei, in relation to the total number of desmin-positive regenerating muscle fibers, was estimated. Results are presented as mean±SD (n = 9). (B) TUNEL-positive nuclei in desmin-positive cells 5 days after injection of either venom or Mtx. To highlight the pattern of spatial heterogeneity, each point corresponds to the density of TUNEL-positive nuclei per area in separate microscopic fields in different areas of the tissue. (C) and (D) Micrographs of muscle tissue sections from mice 5 days after injection of Mtx (C) or *B. asper* venom (D) immunostained for desmin (green fluorescence) and with TUNEL (reddish coloration in nuclei). No TUNEL-positive regenerating fibers, showing centrally-located nuclei, are observed in muscle injected with Mtx, whereas several regenerating fibers present TUNEL-positive nuclei in tissue injected with venom (arrows). Bar represents 50 µm.

As described above, both histological and immunohistochemical analyses evidenced profound differences during the first 7 days after injection between muscles treated with Mtx and those injected with venom. Thus, it is likely that critical events occur during these early stages in the reparative and regenerative scenario. In order to assess whether myogenic and regenerating fibers undergo cell damage, tissue sections were stained with TUNEL, together with Pax7 or desmin, which are markers of activated satellite cells/myoblasts and regenerating muscle cells, respectively [36]. No significant differences were observed in the number of Pax7-positive cells per area, at 3 days, between muscles injected with venom and with Mtx (not shown), thus revealing a similar extent of activation of satellite cells after these treatments. Moreover, there were no significant differences in the percentage of Pax7-positive cells showing positive TUNEL staining at this time interval (venom: 7.3±4.9 %; Mtx: 4.3±2.4 % (mean±SEM, n = 9); p > 0.05). When the percentage of desmin-positive cells having at least one TUNEL-positive nucleus was quantified, no significant differences were observed between treatments at 3, 5 and 7 days, despite a trend showing more TUNEL-positive nuclei in venom-injected muscle at 3 and 5 days (Fig 4A). Examination of tissue sections revealed a noticeable spatial heterogeneity in the pattern of TUNEL staining, especially in muscle injected with venom, since there were areas in which many myonuclei were TUNEL-positive, whereas this staining was lower or largely absent in other areas in venom-injected and, especially in Mtx-injected tissue (Fig 4B, C and D).

### Intramuscular nerves are affected by venom, Mtx and BaP1

**Figure 5:**
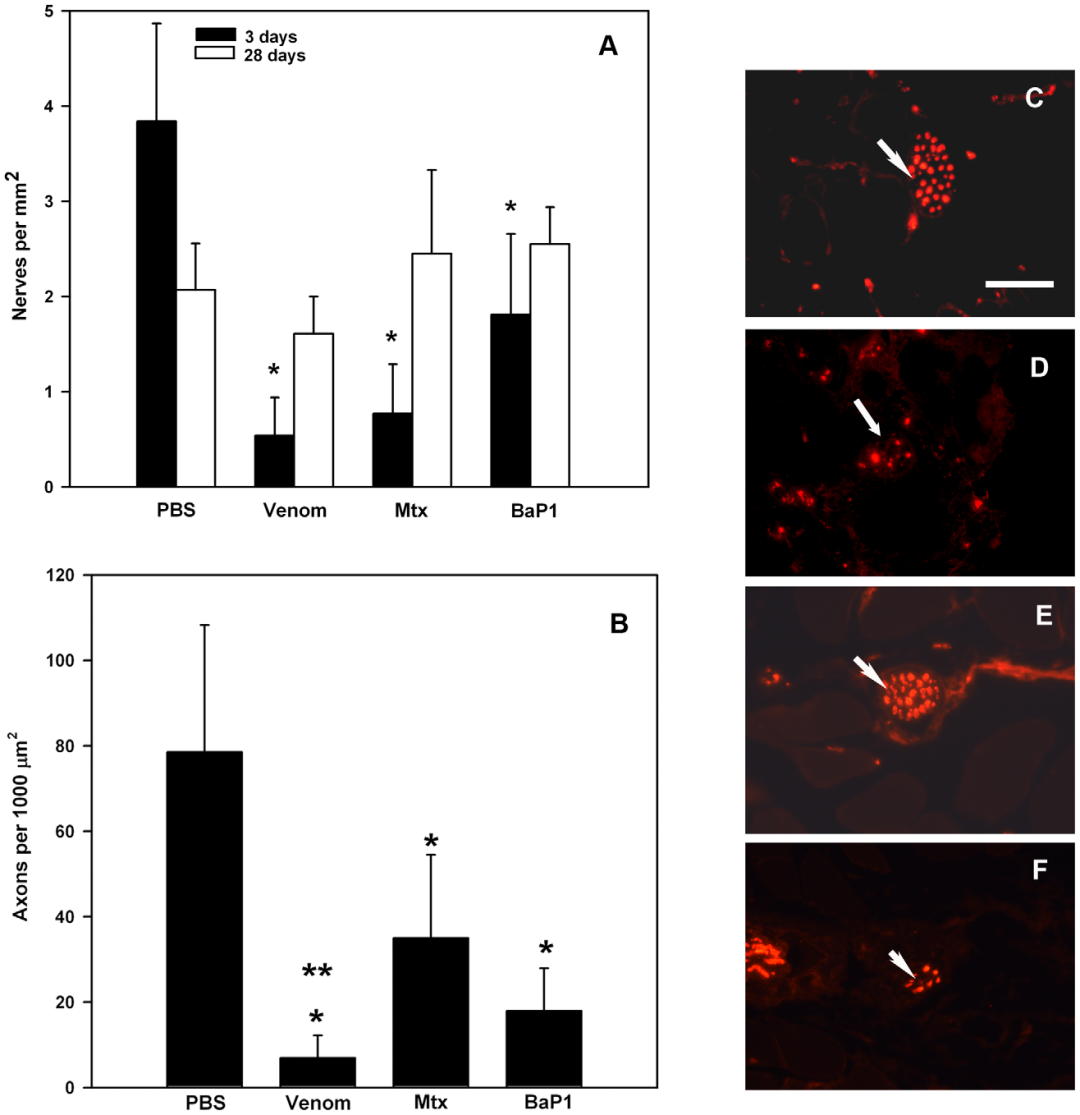
(A) Changes in the density of intramuscular nerves in muscle tissue of mice 3 and 28 days after injection of either PBS, *B. asper* venom, Mtx or BaP1. Axons in nerves were visualized by immunostaining with a mouse anti-human neurofilament protein, as described in Materials and Methods. The number of intramuscular nerves per mm^2^ of tissue area showing at least one immunostained axon was quantified. Results are presented as mean±SD (n = 5). A significant drop (* p < 0.05) in nerves per area was observed at 3 days in samples injected with either venom, Mtx or BaP1, as compared to samples injected with PBS, whereas no differences in the number of nerves per area were detected between treatments at 28 days. (B) Axonal density in intramuscular nerves 28 days after injection of the various agents. The number of axons within each nerve was determined and expressed in terms of axons per nerve area. Results are presented as mean±SD (n = 11). * p < 0.05 when compared with axonal density in control muscles injected with PBS. **p < 0.05 when compared with axonal density in muscles injected with Mtx. (C to F) Light micrograph sections of mouse muscle tissue collected 28 days after injection of (C) PBS, (D) *B. asper* venom, (E) Mtx, and (F) BaP1. Sections were immunostained for neurofilament protein to detect axons in nerves (arrows). Notice the evident drop in the number of axons in samples from tissue injected with venom or BaP1. Bar represents 50 µm.

Since an adequate innervation is a requisite for successful muscle regeneration, the integrity of intramuscular nerves was assessed by immunostaining with anti-neurofilament protein. A similar drop in nerve density was observed at 3 days in tissue injected with either venom, Mtx or BaP1, as compared with tissue injected with PBS (Fig 5A), thus reflecting acute damage to axons. A reduction in the density of nerves in control samples injected with PBS was observed at 28 days, as compared with 3 days, probably reflecting the normal growth of muscle tissue in mice. The number of nerves per area showing immunostained axons in tissue injected with venom, Mtx or BaP1 increased at 28 days, as compared with the same treatments at 3 days, and no difference was detected between control muscle and muscles injected with venom, Mtx or BaP1 at 28 days (Fig 5A). However, a conspicuous difference was noticed in the density of axons within nerves in the various treatments observed in immunostained sections. At 28 days, the density of axons within nerves was significantly lower in all treatments, as compared with muscle injected with PBS (Fig 5 B, C, D, E and F). However, the reduction in axons in venom-treated muscles was more pronounced than in Mtx-and BaP1-injected muscle (Fig 5B).

## Discussion

Local tissue damage in viperid snakebite envenomings is mainly due to the action of myotoxic PLA_2_s and hemorrhagic SVMPs, with the possible involvement of other components such as hyaluronidases and non-hemorrhagic SVMPs [9], [37]–[39]. In turn, skeletal muscle regeneration is a highly orchestrated phenomenon whereby resident cells, phagocytic cells and extracellular matrix interact in a complex scenario. The present work used quantitative experimental procedures that allowed the study of muscle regeneration, microvascular density, fibrosis, inflammatory infiltrate and nerve density, thus providing a greater depth in the analysis of this model of muscle regeneration. One consistent finding was that treatments involving the direct damage to the microvascular network resulting in hemorrhage, i.e. crude venom and BaP1, provoked prominent acute tissue damage followed by a deficient regenerative response associated with insufficient removal of necrotic debris, fibrosis and atrophic regenerative fibers. In contrast, tissue injected with Mtx alone developed acute myonecrosis without hemorrhage, followed by a largely successful regenerative process. These observations agree with previous findings with various snake venoms and toxins evidencing poor regeneration in conditions where microvascular damage, i.e. hemorrhage, occurred [22], [24]–[26], [28], [40].

Our immunohistochemical observations on capillary vessel density corroborated the rapid damage to the microvasculature and the subsequent revascularization process. Therefore, it is likely that the inflammatory reaction that followed acute tissue damage contributed with angiogenic factors, the nature and source of which remain to be investigated. Nevertheless, capillary density remained low during the first days in venom-injected muscle, as compared with control values and with capillary density in Mtx-injected muscle. This observation is relevant because critical events occur in the process of regeneration during those first days, associated with myoblast replication and fusion [13], [36]. Thus, the concept that particular tissue events have to occur at a proper ‘timing’ is of paramount importance in muscle regeneration. In the case of muscle injected with hemorrhagic venom, a deficit in capillary vessel density at critical times during the first days after necrosis is likely to preclude relevant steps in this process, regardless of the fact that there is a successful process of revascularization at later time intervals.

Two main consequences of the acute microvascular damage in this model are: (a) an impairment of the inflammatory infiltrate in areas of widespread microvessel damage, and (b) a reduced supply of oxygen and nutrients to the regenerating tissue in these areas. The drastic damage to the microvasculature is likely to preclude the timely arrival of neutrophils and macrophages in some regions, as shown in this study. This, in turn, may bring at least two implications: First, there is a deficient removal of necrotic debris, a phenomenon evidenced by the presence of remnants of necrotic cells 7 days after venom injection in some tissue sections. Neutrophils and ‘inflammatory’ CD68 macrophages play a key role in necrotic cell removal, and a reduced population of these inflammatory cells in some affected areas precludes the completion of this step [17]. Depletion of neutrophils resulted in a deficient removal of necrotic debris and impaired regeneration after myonecrosis induced by *B. asper* venom and myotoxin in a mouse model [41]. Moreover, depletion of phagocytic macrophages was associated with a slower clearance of necrotic debris in a model of freeze injury [42]. Thus, without a timely removal of necrotic debris by phagocytosis, regeneration is impaired.

On the other hand, phagocytes, particularly macrophages, are known to promote muscle regeneration by synthesizing various mediators in the tissue microenvironment. After removal of necrotic debris, by neutrophils and ‘inflammatory’ CD68 macrophages, a population of ‘anti-inflammatory’ CD163 macrophages invade the tissue 2 to 4 days after injury, releasing anti-inflammatory cytokines, such as IL-10, and factors that promote the proliferation and differentiation of myoblasts, including diverse growth factors and cytokines [13], [17], [43], [44]. The adequate balance in these mediators determines whether myogenesis or fibrogenesis predominates [45]. Furthermore, macrophages are known to prevent myoblasts and myotubes from undergoing apoptosis, through direct cell-cell interactions mediated by a set of adhesion molecules [46]. A deficit in this population of CD163 macrophages in some areas of damaged muscle would jeopardize the provision of growth factors and survival signals.

Another consequence of microvascular damage, in the context of muscle regeneration, is ischemia, with a reduction in the supply of oxygen and nutrients, which are essential requirements for the metabolically-demanding regenerative process [13], [21]. This does not occur in tissue injected with myotoxic PLA_2_s, where capillary density and blood supply are not impaired [21], [22]. The possible effect of microenvironmental factors favouring apoptosis in myogenic cells was assessed by TUNEL staining of Pax7 and desmin-positive cells. No differences occurred in Pax7 cells, thus suggesting that variations in apoptosis of activated satellite cells/myoblasts is not the cause of the different regenerative response between treatments. Moreover, no significant difference was observed in the overall extent of TUNEL staining in desmin-positive cells between muscles treated with either Mtx or venom. Nevertheless, there were areas in venom-injected tissue showing high numbers of TUNEL-positive myonuclei in regenerating fibers, suggesting that myonuclei damage is more prone to occur in some areas, perhaps where the microvasculature is more severely damaged. There are microenvironmental clues known to promote apoptosis, such as ischemia or insufficient survival signals, at this critical window of time in the regenerative process. Apoptotic nuclei have been described in a number of muscle pathologies, such as denervation-induced atrophy and dystrophies [47]–[50]. The observation of tissue areas with abundant TUNEL-positive nuclei in regenerating fibers, *vis-à-vis* the lack of overall quantitative differences between treatments, underscores the heterogeneous nature of tissue damage and repair in this model. This supports the concept that a proper analysis of these phenomena should consider, in addition to the overall quantitative tissue changes, the qualitative assessment of this heterogeneous pattern [51].

Another significant difference between muscle regeneration in tissue injected with venom, BaP1 or Mtx is the abnormally small diameter of regenerating fibers in the former two treatments. This observation may reveal the presence of atrophic fibers, since although the first stages in myoblast replication and fusion do not require an intact nerve supply [13], the further growth of regenerating fibers is dependent on a functional innervation [21], [52]. Quantification of nerves by immunostaining with neurofilament protein revealed a drastic reduction at 3 days. This observation indicates that both Mtx and BaP1, and consequently venom as well, affect the integrity of axons in intramuscular nerves. Despite the fact that *Bothrops* sp myotoxic PLA_2_s do not exert a systemic neurotoxic effect, they are known to affect transmission in nerve-muscle preparations [53], [54], suggesting that direct contact of these myotoxins with nerve terminals may result in axonal damage, as occurs with typical neurotoxic PLA_2_s [55]. Moreover, the products of phospholipids hydrolysis, i.e. lysophospholipid and fatty acids, are capable of affecting the integrity of nerve terminals [56]. Thus, *B. asper* myotoxic PLA_2_s are likely to damage the integrity of axons. Intramuscular nerve axonal damage after injection of *Bothrops* sp venoms has been previously documented [24], [57]. BaP1 also induced nerve damage, indicating that this effect is caused by SVMPs as well. Alternatively, axonal damage after injection of BaP1 and venom might be secondary to ischemia resultant from SVMP-induced microvessel pathology. Nerve damage was followed by axon regeneration, as demonstrated by the increment of structures showing positive immunostaining for neurofilament protein by 28 days. However, although the total number of nerves was similar at this time interval in venom-, BaP1- and Mtx-injected muscles, the density of axons in each nerve was evidently reduced in tissue affected by these three agents, particularly by venom, suggesting that there might be other venom components, in addition to Mtx and BaP1, which may affect axonal regeneration. It is suggested that the limited extent of axonal regeneration in venom and BaP1-treated muscle might be a consequence of the effect of SVMPs on the integrity of the basal lamina of nerves. It has been shown that when such extracellular matrix structure is damaged, reinnervation of muscle is poor [18]. Such SVMP-induced damage to basal lamina may affect various components including agrin, a heparan sulphate proteoglycan that plays a key role in axon growth and the formation of neuro-muscular junctions [18]. In the case of Mtx-induced axonal damage, the preservation of basal lamina is likely to contribute to a more complete reinnervation, albeit even in this case the axonal density within nerves is still lower than in control samples 28 days after injection.

An alternative mechanism explaining the presence of small regenerating fibers in venom-affected muscle may be associated with SVMP-induced damage to the basement membrane surrounding muscle fibers. The integrity of such extracellular matrix scaffold during regeneration is highly relevant, since myoblasts proliferate and fuse within the space delimited by the basement membrane of necrotic fibers, therefore ensuring an orderly restitution of tissue architecture [13], [19]. SVMPs are known to hydrolyze basement membrane components *in vitro* [58], [59] and *in vivo* [59], [60]. When proliferating myoblasts are not confined to the space delimited by the basement membrane, they may proliferate outside such space. In these conditions, fewer cells would undergo fusion and, consequently, small caliber myotubes and myofibers would be generated [13]. Further studies are required to assess the role of SVMP-induced degradation of muscle fiber basement membrane in the process of muscle regeneration. It is hypothesized that the presence of atrophic regenerating fibers in tissue injected with venom is predominantly a consequence of SVMP damage to the basement membrane components of nerves and muscle.

In conclusion, our results show that skeletal muscle regeneration is partially deficient after acute tissue damage induced by the injection of *B. asper* venom and by a hemorrhagic SVMP, whereas a successful regenerative response occurs after myonecrosis induced by a myotoxin which affects muscle fibers but not the microvasculature nor the basement membranes of muscle fibers and nerves. These results support the hypothesis that SVMP-induced basement membrane damage, in microvessels, muscle fibers and nerves, is the main culprit for the poor regenerative outcome in this model. Damage to the microvasculature is likely to induce ischemia and to affect the arrival of phagocytic cells in some necrotic areas. In addition, axonal regeneration is impaired. As a consequence of this complex tissue scenario, fibrosis substitutes muscle tissue in some areas, and the diameter of regenerating fibers is abnormally small. These observations reproduce clinical findings and provide an experimental frame to assess the mechanisms involved in such poor regenerative outcome and to test therapeutic interventions aimed at improving muscle regeneration in people suffering from snakebite envenoming. Moreover, our findings underscore the potential usefulness of inhibiting SVMPs *in situ* after venom injection, in order to prevent tissue damage and to facilitate regeneration. Since similar pathological effects are induced by many viperid snake venoms in different parts of the world, our observations and conclusions may have broader implications in the context of snakebite envenoming.
