# Correction: Poor Regenerative Outcome after Skeletal Muscle Necrosis Induced by *Bothrops asper* Venom: Alterations in Microvasculature and Nerves

**DOI:** 10.1371/annotation/9caa2be8-b5e6-4553-8575-f0b575442172

**Published:** 2011-07-21

**Authors:** Rosario Hernández, Carmen Cabalceta, Patricia Saravia-Otten, Alessandra Chaves, José María Gutiérrez, Alexandra Rucavado

An error was introduced, during processing of this article for publication, in the affiliations of the senior author Rosario Hernández and of Patricia Saravia-Otten. The correct first affiliation is: "Facultad de Ciencias Químicas y Farmacia, Universidad de San Carlos de Guatemala, Guatemala."

